# The Effect of Cigarillo Packaging Characteristics on Young Adult Perceptions and Intentions: An Experimental Study

**DOI:** 10.3390/ijerph18084330

**Published:** 2021-04-19

**Authors:** Cristine D. Delnevo, Michelle Jeong, Ollie Ganz, Daniel P. Giovenco, Erin Miller Lo

**Affiliations:** 1 Rutgers Center for Tobacco Studies, Rutgers Biomedical and Health Sciences, Rutgers University, New Brunswick, NJ 08901, USA; michelle.jeong@rutgers.edu (M.J.); og96@cts.rutgers.edu (O.G.); loer@scts.rutgers.edu (E.M.L.); 2 Department of Sociomedical Sciences, Mailman School of Public Health, Columbia University, New York, NY 10032, USA; dg2984@cumc.columbia.edu

**Keywords:** tobacco regulatory science, tobacco control, communication, cigars, tobacco packaging, product perceptions

## Abstract

Research demonstrates that characteristics of cigarette packaging influence consumer product perceptions, yet the current literature on the impact of cigar packaging is limited. This study aims to examine how different cigarillo packaging features influence young adult cigar smokers’ perceptions. In 2016, we recruited past-year cigar users aged 18–34 from Amazon Mechanical Turk (N = 1260). We utilized a 2 × 2 × 3 × 2 between-subjects factorial design, randomly assigning participants to view one of 24 images of a cigarillo package that varied by brand (Black & Mild vs. Swisher Sweets), brand name (full vs. abbreviated), color (brown vs. green vs. purple), and price promotion (present vs. absent). Participants rated the product on several perceptions and purchase intentions, and they reported on cigar use and demographics. Overall, color and brand name influenced perceptions, but effects varied by brand. For Swisher Sweets, only price promotions influenced perceptions (e.g., taste, use for marijuana); for Black & Mild, all packaging features influenced perceptions (e.g., harshness, tobacco quality), and price promotions increased purchase intentions. Our findings also raise questions that product features may interact with one another, with certain features, such as color, overpowering other attributes. More research is needed to understand the impact of other packaging features, such as warning labels, on product perceptions across a variety of brands.

## 1. Introduction

While cigarette use in the United States (U.S.) has significantly decreased over the past decade, sales and consumption of cigars have steadily increased [1,2,3], as has the variety of cigar brands, sizes, tip styles, and flavors available on the market [3,4]. Flavored cigars in particular have experienced a dramatic growth in sales since 2008 [3,5]–in part due to the federal ban on flavored cigarettes [6] and the rapid proliferation of flavor varieties [4], which drove most of the growth in overall cigar sales [7]. Of the various types of cigars, cigarillos are the most popular [8,9]; in fact, industry reports show cigarillos currently make up more than half of the cigar market [10].

Various data sources point to high rates of cigar use among young adults [9,11,12,13,14]; most notably, the National Survey on Drug Use and Health has consistently shown that the prevalence of past month cigar use is highest among young adults compared to all other age groups [15]. Moreover, cigars are the most common product initiated during young adulthood [15]. Not surprisingly, the majority of young adult cigar smokers report using cigarillos as their primary cigar product [16]. There are many factors that drive cigar popularity among young adults, including flavors that appeal to young people [7,9,17,18], a lack of awareness and misperceptions about the risks of cigar use [19,20], and more affordable pricing that results from differing tax rates and less stringent marketing regulations [21,22]. Another potential yet understudied aspect of cigar popularity is the visual appeal of cigar packaging.

Past studies have highlighted the importance of packaging as a tobacco marketing strategy that can influence brand imagery and consumer perceptions [23,24,25,26,27,28]. Tobacco products, including cigars, have diverse packaging that can explicitly and implicitly communicate different attributes surrounding the product’s flavor, taste, and harshness. Visual presentation, such as the use of color on packaging, can impact consumers’ perceptions about the product itself, including its taste and flavor [29,30]. Given the diversity and abundance of visual components that companies can potentially manipulate on a single package, it is important to recognize and examine how such components can independently influence perceptions, particularly among vulnerable populations, such as young adults. However, much of the current evidence comes from the literature on cigarette packaging, and cigar-specific research is scant.

Similar to research on cigarettes, findings from studies on perceptions of cigar packaging have identified that packaging descriptors and claims (e.g., “natural”) [31,32], branding [32], price promotions [32,33], and colors [31,32,34] are appealing to consumers. Moreover, many appealing cigar packaging characteristics are unique to cigars and not apparent in cigarettes. These include resealable pouches [32,35], small pack sizes [31,32,36], shiny foil packaging [36], and diverse flavors promoted via flavor descriptors and pack imagery [31,32,34]. Nascent research has found that some of these attributes, including flavor descriptors, influence perceptions of harm and addictiveness [34] as well as intentions to use the product [34].

While these studies draw attention to attributes of cigar packaging that appeal to consumers and may influence product perceptions and intentions to use, most relied on qualitative data or experimental studies using fictitious brands or the complete absence of branding and thus have low external validity. Furthermore, there is a need for examination of how perceptions of cigar packaging differ by brand, given the diversity of cigar packaging and varying user profiles across different cigar brands [37]. As such, this study aims to examine how different features of cigarillo packaging influence young adults’ cigarillo perceptions. Specifically, we examine the influence of four packaging characteristics: brand, abbreviation of brand name, color, and presence of price promotions.

## 2. Materials and Methods

### 2.1. Participants

In 2016, we recruited young adult participants from Amazon Mechanical Turk (MTurk), which is a platform that is well-suited for experimental studies and is commonly used to recruit participants for social and behavioral science research [38,39,40]. To be eligible for the study, participants had to be 18–34 years old and had to have used a cigar (i.e., used any type of cigar or cigarillo, smoked a blunt) in the past 12 months. Of the 1279 eligible participants, 19 were dropped due to having duplicate survey entries, completing less than 50% of the survey, having more than one missing value for the experimental questions or failing two or more attention checks. The final sample included 1260 participants.

### 2.2. Procedures

**Figure 1:**
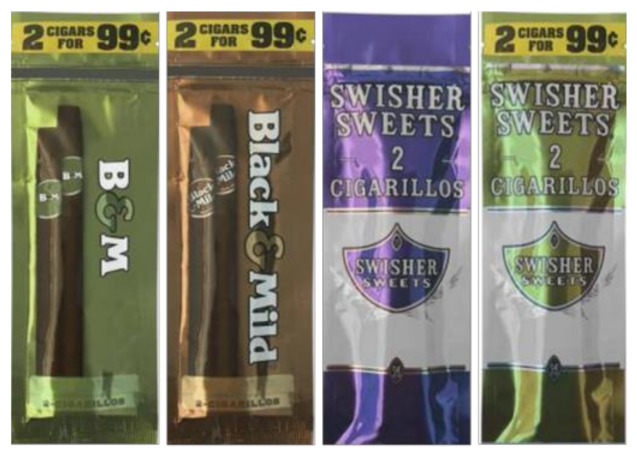
Examples of cigarillo packages from the survey.

We conducted an online experiment with a 2 × 2 × 3 × 2 between-subjects factorial design, in which we randomly assigned participants to view one of 24 images of a resealable, 2-count foil cigarillo package that varied by brand (Black & Mild vs. Swisher Sweets), brand name (full vs. abbreviated name (i.e., B&M, Swisher)), color (brown vs. green vs. purple), and price promotion (present vs. absent). We digitally created the images to manipulate each factor, making sure none of the images explicitly presented flavor names (Figure 1). After viewing the image, participants rated the product on several perceptions. Participants also responded to survey measures assessing cigar use and demographics. The Rutgers Biomedical and Health Sciences Institutional Review Board approved all research procedures for this study.

### 2.3. Measures

We assessed the extent to which participants agreed or disagreed with the following statements: “I think this product might taste good”; “I think this product might smell nice”; “This product looks like it is high-quality tobacco”; “This product looks like it is used to smoke marijuana”; “This product looks like it is fresh”; “This product looks like it is harsh-tasting”; “This product looks like it is harmful to your health.” All responses were on five-point scales ranging from “Strongly disagree” to “Strongly agree.” For analyses, we dichotomized all outcome measures such that 1 = “Strongly agree” and “Agree” and 0 = “Strongly disagree,” “Disagree,” and “Neither agree nor disagree.” We reverse coded responses related to perceptions of the product being “harsh-tasting” and “harmful to your health,” such that the outcomes were “not harsh-tasting” and “not harmful to your health.” Then, respondents were asked about their intention to purchase the product using a modified version of the Juster scale [41]; we asked respondents, “What is the likelihood that you will buy this product in the future?” Responses were on a five-point scale ranging from “Certain or practically certain” to “No chance or almost no chance.” For analyses, we dichotomized all outcome measures such that 1 = “Certain or practically certain” and “Probable” and 0 = “Some possibility,” “Very slight possibility,” and “No chance or almost no chance.”

To assess cigar use, participants were asked, “Do you currently use a [CIGAR TYPE] every day, some days, rarely, or not at all?” for traditional cigars, cigarillos for tobacco, cigarillos for marijuana/blunts and filtered cigars. Respondents were categorized as a user of each product if they reported using the product every day or some days. Given that participants were being exposed to images of Black & Mild and Swisher Sweets packaging, we created a dichotomous Black & Mild and Swisher Sweet brand preference variable (i.e., usually uses/does not usually use), since participants may view their own brand differently, and as such, this could influence their responses. Brand preference was assessed for each cigar type among past-year users of each product with the following question: “What brand of [CIGAR TYPE] do you use most often?” Black & Mild and Swisher Sweets were response choices for both cigarillos and filtered cigars, since both brands sell both cigar types—as such, we used response to the cigarillos and filtered cigar item.

The following demographics were assessed: gender (male, female), age, race (white, Black or African-American, other), ethnicity (Hispanic/Latino, non-Hispanic/Latino), sexual orientation (sexual minority, heterosexual), student status (part/full time student, not a student), highest level of education completed (12th grade or less, some college or greater), employment (full-time, part-time, not currently working), and income ($30,000 or less, greater than $30,000).

### 2.4. Data Analyses

First, we conducted bivariate analyses to confirm that respondent characteristics did not differ by experimental condition. We found that experimental conditions differed by age, gender, and employment; therefore, all multivariable models included these variables as covariates. Next, we used descriptive statistics to characterize the sample in terms of demographics and cigar use behavior. Then, we conducted chi-square tests to estimate the proportion of respondents in each experimental condition who reported agreement with each of the study outcomes. Next, separate multivariable logistic regression models were used to examine the association between brand, brand name, color, and price promotion, and each study outcome among the overall sample and stratified by brand, controlling for age, gender, and employment. Stratified models also controlled for whether the user’s preferred brand was the same as the brand in the given condition (Swisher Sweets or Black & Mild). Lastly, interaction terms were added to all the stratified models to examine the combined effects of the different experimental conditions, above and beyond their main effects and covariates. All statistical tests used a critical alpha of 0.05 and were conducted in Stata/MP 16.1 [42].

## 3. Results

Table 1 presents all participant demographics and tobacco use behaviors. The mean age of participants was 26.5 years, and a little over half (57%) were employed full-time. More than half of the sample was male (63%) and white (75%).

### 3.1. Pack Color

Overall, pack color affected perceptions of the product tasting good, smelling nice, and not being harsh (Table 2). However, analyses stratified by brand showed that there was no main effect of package color for Swisher Sweets (Table 3). Interestingly for Swisher Sweets, the color condition interacted with the brand name condition on the perception that the product was fresh; use of the abbreviated brand name (i.e., Swisher) reduced perceptions of freshness only when the pack was brown; green and purple packs were unaffected by brand name abbreviation (*p* < 0.05).

On the other hand, pack color increased several favorable perceptions of Black & Mild products (Table 4). Specifically, the purple pack was more likely to be perceived as smelling nice (*p* < 0.05), the green pack was more likely to be perceived as being used for marijuana (*p* < 0.05), and both the green and purple packs were more likely to be perceived as tasting good, being fresh, and not being harsh, compared to the brown pack.

### 3.2. Price Promotion

Although price promotion did not affect perceptions or intentions in the overall sample (Table 2), it affected different perceptions when stratified by brand. For Swisher Sweets, the presence of a price promotion reduced perceptions that the product was used for marijuana (*p* < 0.05) (Table 3). For Black & Mild, a price promotion reduced perceptions that the product tasted good and was high quality (both *p*s < 0.05), but it increased intentions to purchase (OR = 1.90, *p* < 0.01) (Table 4).

### 3.3. Brand Name

Use of the abbreviated brand name reduced favorable perceptions of the product compared to use of the full brand name, and it was associated with lower perceptions of the product tasting good, smelling nice, and not being harsh (all *p*s < 0.01) (Table 2). When examining these findings by brand, there were some differences for Black & Mild but not Swisher Sweets. When the brand was Black & Mild, use of the abbreviated brand name (i.e., B&M) reduced perceptions of the product smelling nice (*p* < 0.01), being high quality tobacco (*p <* 0.05), and not being harsh (*p <* 0.001) (Table 4). There was a negative effect of the abbreviated brand name on intention to purchase, but it was only borderline significant (*p* = 0.05).

## 4. Discussion

The findings of this study illustrate that packaging plays an important role in shaping young adult cigar users’ perceptions of cigar products. It is worth noting that packaging elements such as color can particularly appeal to younger adults who may be experimenting with tobacco use. Our findings align with previous studies that have shown the effect of packaging on various product perceptions [31,32,33,34,35]. Favorable perceptions, such as the belief that the product tastes good, is not harsh, and is not harmful, have been linked with various behavioral outcomes, such as intentions to purchase.

Our findings showed color to be an influential packaging feature. For Black & Mild, we found that colorful packaging (i.e., purple and green) was associated with more favorable perceptions of the product compared to brown packaging. Furthermore, our interaction models showed that for Swisher Sweets packaging, color overpowered the impact of another element of the packaging: brand logo. Our findings build on the existing literature pertaining to the effects of color in the context of other tobacco products, including cigarillos [31]. Consumers assess a product based on the packaging in a process called sensation transference [30]. For example, people associate certain colors with certain flavors and flavor attributes. Our findings, which show that colorful packaging impacts perceptions of taste, smell, freshness, and harshness even without explicit flavor descriptors, suggest that color conveys similar meanings in the context of cigarillo packaging. As such, this raises a critical question as to whether products that undergo changes to the color of the packaging should be considered new tobacco products under the U.S. Food and Drug Administration (FDA) Center for Tobacco Products’ deeming rule, rather than equivalent to the original product, due to the differential impact they have on perceptions [31]. Currently, changes to the design of the package are not considered for FDA substantial equivalence reviews.

Price promotion was the only packaging element that had direct associations with purchase intentions. Specifically, the presence of a price promotion increased intentions to purchase Black & Mild products, despite being associated with perceptions of worse taste and lower quality of the tobacco. Similarly, previous studies have found that price promotions on the packaging drove interest in the product [33].

Use of the abbreviated brand name (B&M) reduced favorable perceptions of the product and, importantly, it increased perceptions of harshness, compared to use of the full brand name (Black & Mild). This points to the potential positive influence of the word “Mild” in the brand name, which is undoubtedly important to Altria, the owners of Black & Mild. With the passing of the 2009 Family Smoking Prevention and Tobacco Control Act (TCA), the FDA was granted the authority to regulate tobacco products. The TCA banned the use of misleading descriptors that communicated reduced harm claims, including “light” and “mild.” Initially, this only included cigarettes, roll-your-own tobacco, and smokeless tobacco, but it was extended to include all tobacco products, including cigars, in 2016 under the deeming rule. Shortly after the deeming rule, Altria filed a lawsuit against the FDA, alleging that removing “mild” from their Black & Mild cigar products “terminates this iconic brand name on the bare supposition that the word mild impermissibly communicates to consumers that Black & Mild products are safer than other cigars and pipe tobacco” [43]. Shortly after, the lawsuit was dropped and the chairman, president and CEO of Altria Group stated during the company’s second-quarter earnings call that FDA indicated that they did not, at that time, plan to enforce the ban on “mild” in regard to Black & Mild [44]. Our findings show that the allowance of “mild” in the Black & Mild name, despite being a banned term for communicating misperceptions of harm, does indeed influence product perceptions related to harshness.

This study has limitations. First, the images used in this study were manipulated in terms of the various packaging elements. However, in an effort to closely mimic what consumers see in the marketplace, we used existing brands, improving external validity compared to past studies. This study also uses a convenience sample, which may not be generalizable to all young adults who use cigars in the U.S. However, research shows that experimental findings obtained via Amazon Mechanical Turk are comparable to findings from national probability samples [38].

## 5. Conclusions

Our study demonstrates that cigarillo packaging characteristics influence young adult consumers’ product perceptions, including their perceptions of taste, smell, harshness, and the quality of tobacco. Our findings also raise questions that product features may interact with one another, with certain features, such as color, overpowering other attributes. In addition to the main effects of color on various favorable product perceptions, our finding that the abbreviated brand logo only influenced consumer perceptions when the packaging was brown, demonstrates that color plays an important role in cigarillo packaging not only in what it directly communicates to consumers but in how it influences the impact of other product features. While this study did not include a warning label condition, future studies should examine how color may affect the impact of warning labels on consumer perceptions. Given that the cigarillo marketplace is diverse and constantly in flux, there is a need for continued research on the impact of cigarillo packaging features on consumer perceptions, beyond the brands and attributes explored in this paper.

## Figures and Tables

**Table 1:** Demographics and cigar use characteristics (n = 1260).

Demographics	% (n)
Gender	
Male	63.1 (795)
Female	36.9 (465)
Age (mean (SD))	26.5 (4.1)
Race	
White	75.4 (949)
Black/African American	12.3 (155)
Other	12.3 (155)
Ethnicity	
Hispanic/Latino	10.2 (128)
Non-Hispanic/Latino	89.8 (1132)
Sexual orientation	
Sexual minority	19.1 (241)
Heterosexual	80.9 (1019)
Current college student (part- or full-time)	32.3 (407)
Education completed	
12th grade or less	11.3 (143)
Some college or greater	88.6 (1117)
Employment	
Full-time	57.1 (720)
Part-time	25.1 (316)
Not currently working	17.8 (224)
Income	
$30,000 or less	57.9 (707)
Greater than $30,000	42.1 (514)
Cigar use ^α^	
Current traditional cigar use *	19.0 (239)
Current cigarillo use (tobacco) *	16.1 (203)
Current cigarillo use (marijuana)/blunt user *	29.0 (365)
Current filtered cigar use*	14.4 (182)
Brand used most often ^α^	
Black & Mild	31.2 (393)
Swisher Sweets	19.4 (245)

^α^ Not mutually exclusive groups, * Defined as every day or some days among past 12-month users.

**Table 2:** Product Perceptions Among the Study Population and Adjusted Logistic Regression Models.

	Taste Good ^a^	Smell Nice ^a^	High Quality Tobacco ^a^	Used for Marijuana ^a^	Fresh ^a^	Not Harsh ^a^	Not Harmful ^a^	Intention to Purchase ^b^
	% (n)	% (n)	% (n)	% (n)	% (n)	% (n)	% (n)	% (n)
Abbreviated Logo & Brand	49.4 (311)	62.4 (394)	22.7 (143)	69.7 (440)	51.3 (321)	29.0 (183)	7.1 (45)	27.3 (172)
Full Logo & Brand	57.2 (360)	70.9 (445)	25.5 (160)	70.9 (446)	55.8 (351)	37.5 (235)	8.3 (52)	31.0 (195)
Color: Brown	46.6 (207)	63.0 (279)	22.4 (99)	67.1 (298)	49.3 (218)	26.0 (115)	6.3 (28)	27.2 (121)
Color: Green	55.2 (217)	66.9 (263)	25.9 (102)	75.3 (296)	57.0 (224)	36.1 (142)	8.4 (33)	30.5 (120)
Color: Purple	58.5 (247)	70.2 (297)	24.2 (102)	69.0 (292)	54.8 (230)	38.2 (161)	8.5 (36)	29.8 (126)
Price Promotion Present	52.3 (312)	65.1 (388)	22.4 (134)	69.7 (416)	54.6 (325)	32.6 (194)	8.0 (48)	31.5 (188)
Price Promotion Absent	54.2 (359)	68.0 (451)	25.6 (169)	70.9 (470)	52.6 (347)	33.8 (224)	7.4 (49)	27.0 (179)
Brand: Black & Mild	47.3 (289)	61.0 (373)	24.1 (147)	62.5 (382)	48.8 (297)	28.2 (172)	6.4 (39)	20.1 (123)
Brand: Swisher Sweets	58.9 (382)	71.9 (466)	24.1 (156)	77.7 (504)	58.0 (375)	38.0 (246)	8.9 (58)	37.6 (244)
	aOR (95% CI)	aOR (95% CI)	aOR (95% CI)	aOR (95% CI)	aOR (95% CI)	aOR (95% CI)	aOR (95% CI)	aOR (95% CI)
Abbreviated Logo & Brand	0.71 (0.57, 0.89) **	0.68 (0.53, 0.86) **	0.84 (0.64, 1.09)	0.95 (0.74, 1.22)	0.84 (0.67, 1.06)	0.67 (0.53, 0.86) **	0.83 (0.55, 1.27)	0.82 (0.64, 1.06)
Full Logo & Brand	Reference	Reference	Reference	Reference	Reference	Reference	Reference	Reference
Color: Brown	Reference	Reference	Reference	Reference	Reference	Reference	Reference	Reference
Color: Green	1.35 (1.02. 1.78) *	1.12 (0.84, 1.50)	1.22 (0.88, 1.68)	1.45 (1.06, 1.97)	1.31 (0.99, 1.73)	1.54 (1.14, 2.08) **	1.34 (0.79, 2.28)	1.09 (0.80, 1.48)
Color: Purple	1.59 (1.21, 2.10) **	1.36 (1.02, 1.82) *	1.12 (0.81, 1.54)	1.08 (0.81, 1.45)	1.21 (0.92, 1.59)	1.74 (1.30, 2.34) ***	1.38 (0.82, 2.31)	1.07 (0.79, 1.44)
Price Promotion Present	0.88 (0.70, 1.10)	0.83 (0.65, 1.06)	0.82 (0.63, 1.06)	0.95 (0.74, 1.22)	1.04 (0.83, 1.31)	0.90 (0.71, 1.15)	1.06 (0.70, 1.61)	1.23 (0.96, 1.58)
Price Promotion Absent	Reference	Reference	Reference	Reference	Reference	Reference	Reference	Reference
Brand: Black & Mild	Reference	Reference	Reference	Reference	Reference	Reference	Reference	Reference
Brand: Swisher Sweets	1.57 (1.26, 1.97) ***	1.63 (1.28, 2.07) ***	0.98 (0.75, 1.27)	2.05 (1.60, 2.63) ***	1.44 (1.15, 1.80) **	1.51 (1.19, 1.93) **	1.40 (0.91, 2.14)	2.36 (1.83, 3.05) ***

^a^ Response options were collapsed: strongly agree/agree v. neutral/disagree/strongly disagree; ^b^ Response options were categorized as certain/practically certain and probable vs. some possibility, very slight possibility, no chance/almost no chance. * *p* < 0.05; ** *p* < 0.01; *** *p* < 0.001. Note: all multivariable models control for age, gender and employment.

**Table 3:** Adjusted logistic regression models for product perceptions: Swisher Sweets.

	Taste Good ^a^	Smell Nice ^a^	High Quality Tobacco ^a^	Used for Marijuana ^a^	Fresh ^a^	Not Harsh ^a^	Not Harmful ^a^	Intention to Purchase ^b^
	aOR (95% CI)	aOR (95% CI)	aOR (95% CI)	aOR (95% CI)	aOR (95% CI)	aOR (95% CI)	aOR (95% CI)	aOR (95% CI)
Abbreviated Logo & Brand	0.73 (0.53, 1.00)	0.82 (0.58, 1.16)	1.05 (0.73, 1.52)	0.71 (0.49, 1.04)	0.84 (0.61, 1.15)	0.85 (0.62, 1.18)	0.90 (0.52, 1.56)	0.95 (0.68, 1.33)
Full Logo & Brand	Reference	Reference	Reference	Reference	Reference	Reference	Reference	Reference
Color: Brown	Reference	Reference	Reference	Reference	Reference	Reference	Reference	Reference
Color: Green	1.04 (0.70, 1.54)	0.94 (0.62, 1.44)	1.32 (0.83, 2.09)	1.23 (0.76, 1.99)	0.90 (0.61, 1.33)	1.33 (0.89, 1.99)	1.85 (0.91, 3.77)	1.07 (0.71, 1.62)
Color: Purple	1.46 (0.98, 2.16)	1.18 (0.77, 1.81)	1.32 (0.83, 2.09)	0.83 (0.53, 1.32)	0.89 (0.60, 1.31)	1.44 (0.96, 2.15)	1.56 (0.75, 3.22)	1.23 (0.81, 1.85)
Price Promotion Present	1.13 (0.82, 1.55)	0.95 (0.67, 1.35)	1.00 (0.70, 1.45)	0.66 (0.45, 0.95) *	1.14 (0.83, 1.57)	1.01 (0.73, 1.39)	1.50 (0.86, 2.60)	0.98 (0.70, 1.37)
Price Promotion Absent	Reference	Reference	Reference	Reference	Reference	Reference	Reference	Reference
Uses another brand	Reference	Reference	Reference	Reference	Reference	Reference	Reference	Reference
Uses Swisher Sweets	1.98 (1.30, 3.00) **	1.60 (1.01, 2.54) *	1.76 (1.15, 2.70) **	0.67 (0.43, 1.05)	1.68 (1.11, 2.52) *	1.61 (1.09, 2.38) *	1.41 (0.75, 2.66)	4.10 (2.73, 6.14) ***

^a^ Response options were collapsed: strongly agree/agree v. neutral/disagree/strongly disagree; ^b^ Response options were categorized as certain/practically certain and probable vs. some possibility, very slight possibility, no chance/almost no chance. * *p* < 0.05; ** *p* < 0.01; *** *p* < 0.001 Note: all multivariable models control for age, gender and employment.

**Table 4:** Adjusted logistic regression models for product perceptions: Black & Mild.

	Taste Good ^a^	Smell Nice ^a^	High Quality Tobacco ^a^	Used for Marijuana ^a^	Fresh ^a^	Not Harsh ^a^	Not Harmful ^a^	Intention to Purchase ^b^
	aOR (95% CI)	aOR (95% CI)	aOR (95% CI)	aOR (95% CI)	aOR (95% CI)	aOR (95% CI)	aOR (95% CI)	aOR (95% CI)
Abbreviated Logo & Brand	0.72 (0.51, 1.01)	0.58 (0.41, 0.82) **	0.66 (0.45, 0.98) *	1.17 (0.84, 1.64)	0.87 (0.63, 1.21)	0.49 (0.33, 0.71) ***	0.72 (0.37, 1.40)	0.66 (0.43, 1.00)
Full Logo & Brand	Reference	Reference	Reference	Reference	Reference	Reference	Reference	Reference
Color: Brown	Reference	Reference	Reference	Reference	Reference	Reference	Reference	Reference
Color: Green	1.68 (1.11, 2.54) *	1.22 (0.81, 1.85)	1.03 (0.65, 1.64)	1.67 (1.10, 2.54) *	1.78 (1.19, 2.67) **	1.67 (1.04, 2.67) *	0.83 (0.35, 1.97)	0.90 (0.54, 1.50)
Color: Purple	1.79 (1.20, 2.67) **	1.55 (1.04, 2.31) *	0.97 (0.61, 1.53)	1.27 (0.86, 1.88)	1.61 (1.09, 2.37) *	2.31 (1.47, 3.63) ***	1.29 (0.61, 2.74)	0.89 (0.55, 1.46)
Price Promotion Present	0.65 (0.46, 0.91)*	0.72 (0.51, 1.01)	0.66 (0.45, 0.97) *	1.26 (0.90, 1.76)	0.95 (0.68, 1.32)	0.77 (0.53, 1.13)	0.67 (0.34, 1.33)	1.92 (1.26, 2.91) **
Price Promotion Absent	Reference	Reference	Reference	Reference	Reference	Reference	Reference	Reference
Uses another brand	Reference	Reference	Reference	Reference	Reference	Reference	Reference	Reference
Uses Black & Mild	2.77 (1.91, 4.02) ***	1.92 (1.31, 2.83) **	1.76 (1.18, 2.64) **	1.01 (0.70, 1.46)	1.62 (1.13, 2.33) **	2.55 (1.73, 3.76) ***	0.54 (0.23, 1.26)	2.79 (1.83, 4.26) ***

^a^ Response options were collapsed: strongly agree/agree v. neutral/disagree/strongly disagree; ^b^ Response options were categorized as certain/practically certain and probable vs. some possibility, very slight possibility, no chance/almost no chance. * *p* < 0.05; ** *p* < 0.01; *** *p* < 0.001 Note: all multivariable models control for age, gender and employment.

## Data Availability

Data are not available in a repository. Data may be requested from Cristine Delnevo (ORCID 0000-0001-9597-4307) and should include a plan for its use. Data will be available to qualified researchers after the main findings are published in a peer-reviewed journal. All data sharing will comply with local, state, and federal laws and regulations and may be subject to appropriate human subjects institutional review board approvals.
